# B Cell Compartmentalization in Blood and Cerebrospinal Fluid of HIV-Infected Ugandans with Cryptococcal Meningitis

**DOI:** 10.1128/IAI.00779-19

**Published:** 2020-02-20

**Authors:** Samuel Okurut, David B. Meya, Freddie Bwanga, Joseph Olobo, Michael A. Eller, Fatim Cham-Jallow, Paul R. Bohjanen, Harsh Pratap, Brent E. Palmer, Katharine H. Hullsiek, Yukari C. Manabe, David R. Boulware, Edward N. Janoff

**Affiliations:** a Research Department, Infectious Diseases Institute, Makerere University, Kampala, Uganda; b Department of Microbiology, School of Biomedical Sciences, College of Health Sciences, Makerere University, Kampala, Uganda; c Department of Medicine, School of Medicine, College of Health Sciences, Makerere University, Kampala, Uganda; d Laboratory Department, Makerere University Walter Reed Project, Kampala, Uganda; e Mucosal and Vaccine Research Program Colorado, Department of Medicine, University of Colorado Denver, Aurora, Colorado, USA; f Denver Veterans Affairs Medical Center, Denver, Colorado, USA; g U.S. Military HIV Research Program, Walter Reed Army Institute of Research, Silver Spring, Maryland, USA; h Henry M. Jackson Foundation for the Advancement of Military Medicine, Bethesda, Maryland, USA; i Division of Infectious Diseases, Department of Medicine, John Hopkins University School of Medicine, Baltimore, Maryland, USA; j Department of Immunology and Molecular Biology, School of Biomedical Sciences, College of Health Sciences, Makerere University, Kampala, Uganda; k Division of Infectious Diseases and International Medicine, Department of Medicine, University of Minnesota, Minneapolis, Minnesota, USA; Tulane School of Medicine

**Keywords:** B cell subsets, activation, plasmablasts/plasma cells, PD-1, HIV, cryptococcal meningitis, survival, B cell activation, blood, HIV coinfection, PD-1 expression, cerebrospinal fluid

## Abstract

Activated B cells modulate infection by differentiating into pathogen-specific antibody-producing effector plasmablasts/plasma cells, memory cells, and immune regulatory B cells. In this context, the B cell phenotypes that infiltrate the central nervous system during human immunodeficiency virus (HIV) and cryptococcal meningitis coinfection are ill defined. We characterized clinical parameters, mortality, and B cell phenotypes in blood and cerebrospinal fluid (CSF) by flow cytometry in HIV-infected adults with cryptococcal (*n* = 31) and noncryptococcal (*n* = 12) meningitis and in heathy control subjects with neither infection (*n* = 10). Activation of circulating B cells (CD21^low^) was significantly higher in the blood of subjects with HIV infection than in that of healthy controls and greater yet in matched CSF B cells (*P* < 0.001). Among B cell subsets, elevated frequencies of memory and plasmablasts/plasma cells most clearly distinguished the CSF from blood compartments. With cryptococcal meningitis, lower frequencies of expression of the regulatory protein programmed death-1 (PD-1) on plasmablasts/plasma cells in blood (median, 7%) at presentation were associated with significantly decreased 28-day survival (29% [4/14 subjects]), whereas higher PD-1 expression (median, 46%) characterized subjects with higher survival (88% [14/16 subjects]). With HIV infection, B cell differentiation and regulatory markers are discrete elements of the circulating and CSF compartments with clinical implications for cryptococcal disease outcome, potentially due to their effects on the fungus and other local immune cells.

## Abstract (precis)

Activated B cells modulate infection by differentiating into pathogen-specific antibody-producing effector plasmablasts/plasma cells, memory cells, and immune regulatory B cells. In this context, the B cell phenotypes that infiltrate the central nervous system during human immunodeficiency virus (HIV) and cryptococcal meningitis coinfection are ill defined.

## INTRODUCTION

Meningitis caused by the encapsulated fungus Cryptococcus neoformans is a leading cause of death among human immunodeficiency virus (HIV)-infected immune-suppressed patients in sub-Saharan Africa, accounting for 15% of their deaths worldwide (1). Despite frequent exposure to the yeast in the environment, cryptococcal infection is very rare in healthy individuals. Immune status is a critical determinant of risk for fatal cryptococcosis. Type 1 helper T cells may contain primary infection in the lungs as a cornerstone of protection among healthy persons (with ≈800 to 1,200 circulating CD4^+^ T cells/μl) (2). However, most HIV-infected patients present with cryptococcal meningitis at a very advanced state of immunosuppression, with CD4^+^ T cell counts of <50 cells/μl (1).

B cells contribute to the development of a competent immune system by inducing naive T cell activation, generating and maintaining serological memory, and regulating immune responses in health and in disease (3, 4). In animal models, B cells produce antibodies against the cryptococcal polysaccharide capsule and other fungal antigens (5, 6) that may attenuate infection and mediate fungal clearance (7). Specific antibodies may support opsonization and killing of the organism by phagocytes (8, 9), neutralization of fungal virulence factors (10), or direct antibody-mediated toxicity and interference with fungal metabolism (7). B cells can produce either proinflammatory (e.g., interleukin-6 [IL-6], tumor necrosis factor alpha [TNF-α], and gamma interferon [IFN-γ]) (11) or anti-inflammatory (e.g., IL-10) cytokines. IL-10-producing regulatory B cells, including plasma cells, modulate the activity of other immune cells in the local environment (4) as may B cells expressing surface immunomodulatory molecules, such as programmed death-1 (PD-1) (12, 13).

The contribution of pathogen-specific antifungal responses can be compromised during HIV-1 infection by polyclonal B cell activation and attenuated humoral responses to primary and recall antigens (14). Both *Cryptococcus* and HIV may have profound influences on B cell activation and differentiation and their effector and regulatory roles in the central nervous system (CNS) where most fatal cryptococcal disease occurs (15). To elucidate B cell signatures in AIDS-related cryptococcosis, we determined B cell phenotypes, activation, and differentiation in blood and in cerebrospinal fluid (CSF) among persons with HIV with cryptococcal and noncryptococcal meningitis and among HIV-negative healthy control subjects with neither infection and the association of these variables with survival.

(This work was presented in part at the Keystone Symposia on HIV Vaccines (X5) conference joint with the Golden Anniversary of B Cell Discovery Meeting in Banff Springs, Banff, Alberta, Canada, 22 to 27 March 2015 [16], and at the EMBO-AIDS related mycoses workshop in Cape Town, South Africa, 13 to 15 July 2016 [17].)

## RESULTS

### Subjects and mortality in HIV-associated meningitis coinfections.

Age and gender did not differ significantly among the 3 study groups (Table 1). Circulating CD4^+^ T cell numbers were low in all HIV-infected subjects tested. CSF protein levels were similar among those with cryptococcal and noncryptococcal meningitis. Although the Glasgow coma score was abnormal in only a quarter of subjects with cryptococcosis (<15 points), 28-day mortality was high.

**TABLE 1:** Baseline characteristics of HIV-infected participants with cryptococcal meningitis or noncryptococcal meningitis and healthy control subjects^*a*^

Group	Cryptococcal meningitis		Noncryptococcal meningitis		Healthy controls		*P* value
	No.	Median (IQR) or no. (%)	No.	Median (IQR) or no. (%)	No.	Median (IQR) or no. (%)	
Total no.	31		12		10		
Clinical parameters							
Age (yr)	31	38 (32–41)	12	39 (35–45)	8	39 (36.5–46.5)	0.81
Female gender	31	8 (25.8)	12	6 (50)	8	4 (50.0)	0.13
Weight (kg)	15	54 (50–58.0)	3	60 (51.0–74.0)			0.31
CD4^+^ T cells/μl (normal, 800 to 1,200)	22	13.5 (6–46.3)	7	53.0 (7.0–279.0)			0.3
Currently on ART	31	2 (6.5)	12	0 (0.0)			
Currently receiving TB therapy	31	1 (3.2)	12	1 (8.3)			0.48
Glasgow coma score of <15	30	7 (23.3)	12	7 (58.3)			0.03
CSF parameters							
Quantitative cryptococcal CFU/ml (log_10_)	30	4.6 (4.1–5.3)	12	0 (0–0)			
Sterile cryptococcal cultures	30	1 (3.3)	12	12 (100.0)			
Opening pressure (mm/H_2_O)	25	259 (160–370)	11	230 (128–278)			0.32
Opening pressure of >250 mm/H_2_O	25	12 (48.0)	11	3 (27.3)			0.25
Total WBC counts (cells/μl)	28	17.5 (4.0–112.5)	11	65 (4.0–320.0)			0.45
Total WBC counts of >5 (cells/μl)	28	15 (53.6)	11	7 (63.6)			0.57
CSF protein (mg/dl)	30	59 (20.0–97.0)	12	95.5 (43–187.0)			0.16

a Statistics used to generate the tabulated values were as follows: chi-square test for comparing proportions between groups, and Kruskal-Wallis test for comparing medians among groups. TB, tuberculosis; WBC, white blood cells; IQR, interquartile range.

Among all HIV-infected subjects with known outcomes, 42.9% (15/35) died in this period, including 40% (12/30; deaths) of those with cryptococcal meningitis. Among subjects with cryptococcal meningitis, median survival time was 10 days (95% confidence interval [CI], 4 to 19 days) for those dying by 28 days and 50 days (95% CI, 32 to 99 days) for those dying after 28 days. Median survival was 19 days (95% CI, 9 to 30 days) for 4 subjects with Mycobacterium tuberculosis meningitis. One subject with meningitis of unknown cause died in 19 days.

### Overall B cell frequency and activation in blood and CSF among subjects with cryptococcosis.

**FIG 1:**
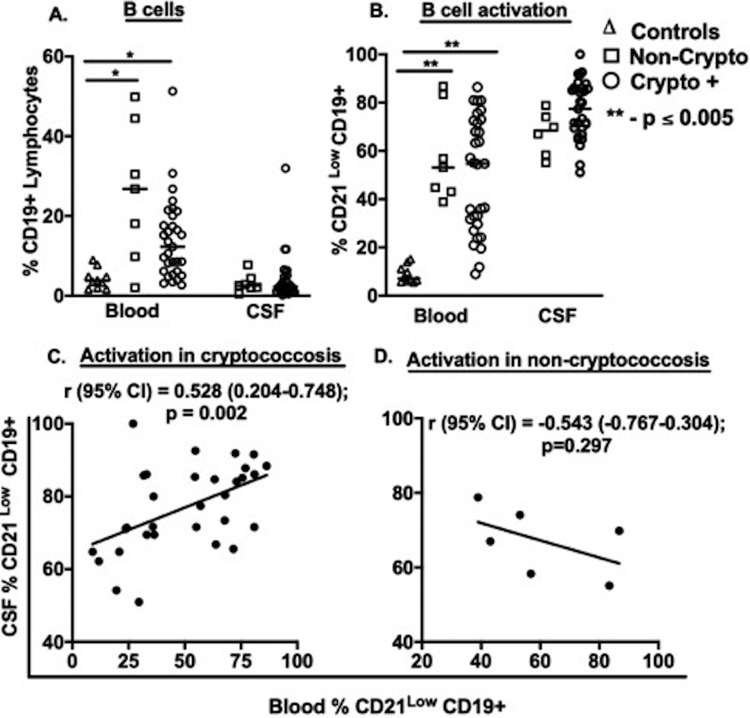
B cell frequency and activation in blood and in cerebrospinal fluid. Frequency of CD19^+^ B cells among lymphocytes (A) and B cell activation (CD21^low^) by flow cytometry (B to D). Samples were collected at presentation from healthy control subjects (blood samples; *n* = 10) and HIV-infected subjects with cryptococcosis (blood [*n* = 31] and matched CSF [*n* = 31] samples) or noncryptococcal meningitis (blood [*n* = 7] and CSF [*n* = 6] samples). Values were compared using Mann-Whitney U test or using Kruskal-Wallis and Spearman’s correlation coefficient. Horizontal bars indicate median values, and asterisks show statistically significant results for *P* values of <0.05.

The CD19^+^ B cells represented a greater proportion of circulating lymphocytes in blood among HIV-infected subjects with low CD4^+^ T cells than among healthy controls (median, 12% in cryptococcosis, 27% in noncryptococcosis, and 4% in healthy controls; analysis of variance [ANOVA], *P* < 0.001) (Fig. 1A). With HIV infection, B cells represented a higher proportion of lymphocytes in blood than in CSF among cryptococcosis subjects (median, 12% versus 2.3%, respectively; *P* < 0.001) and among noncryptococcosis subjects (median, 27% versus 2.6%, respectively; *P* = 0.011).

The B cell activation was significantly higher in both HIV-infected groups than in healthy controls in blood (median, 55% and 53% versus 7%, respectively; *P* < 0.03) and higher yet in CSF (68% and 77%) (Fig. 1B). Among cryptococcosis subjects, B cell activation in CSF positively correlated with that in blood (Fig. 1C) but not among noncryptococcal subjects (Fig. 1D).

### B cell subsets and activation in blood and CSF.

**FIG 2:**
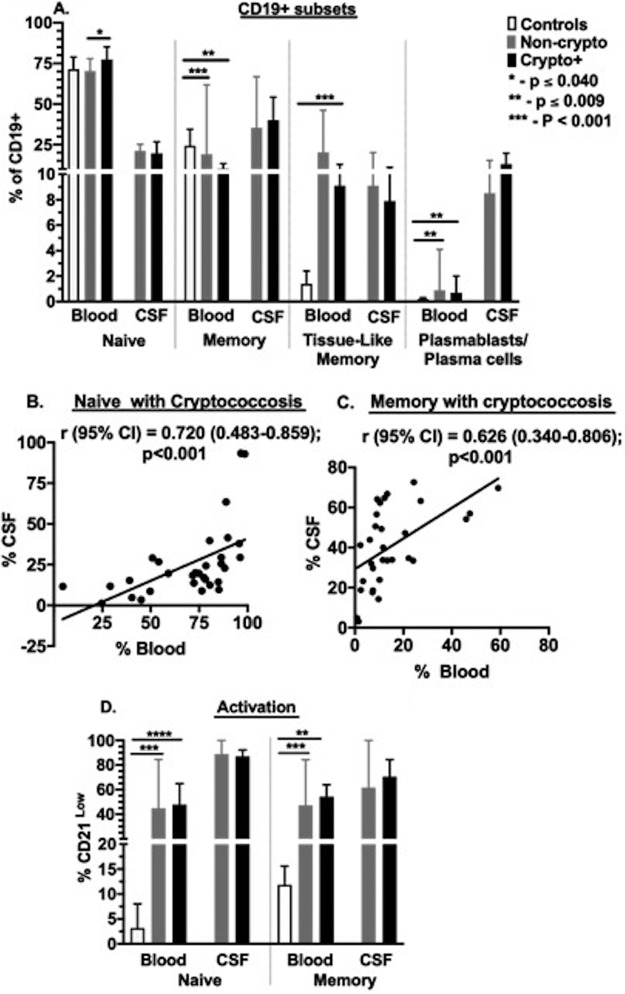
Distribution of B cell subsets and activation in blood and CSF. Frequency of B cell subsets among CD19^+^ lymphocytes by flow cytometry (defined in Table S1 in the supplemental material). (A) Subset distribution. (B and C) Correlation of naive and memory B cells. (D) Activation of naive and memory B cells. Control samples (blood; *n* = 10), cryptococcosis subject samples (blood and CSF; *n* = 31), and noncryptococcosis subject samples (blood and CSF; *n* = 7). Results are shown as medians (95% CI). Values are compared using either Mann-Whitney U test or Kruskal-Wallis test. Asterisks show statistically significant results for *P* values of <0.05.

Circulating B cells in blood showed distinct differences in subset distribution and activation (see Table S2 in the supplemental material). Naive B cells predominated in blood in all groups (Fig. 2A). Memory cells in blood were significantly lower among both HIV-infected groups than in healthy control subjects. Tissue-like memory cells were over 5-fold higher with HIV infection than in healthy controls. Plasmablasts/plasma cells (PB/PCs), although a minority population in blood, were overrepresented with HIV infection (Fig. 2A).

In the CSF, B cells showed a more differentiated phenotype (Table S2), with naive cells representing only about a quarter of cells compared with the majority in blood in all groups (Fig. 2A); these proportions correlated in the two compartments (Fig. 2B). Memory cells were also prominent in the CSF, accounting for up to half of B cells, and also correlated with those in blood (Fig. 2C), suggesting trafficking between the two compartments. Plasmablasts/plasma cell frequencies in CSF greatly exceeded those in blood in HIV-infected subjects with (median, 13% versus 0.7%; *P* < 0.001) and without (median, 9% versus 1%; *P* = 0.008) cryptococcosis (Fig. 2A).

In addition to subset differences between circulating B cells, patients with HIV demonstrated significantly higher levels of activation in both naive and memory cells than did healthy controls (Fig. 2D). Activation was greater yet in B cells in the CSF, particularly among naive cells, as well as among memory cells. These data indicate that B cells that traffic and localize to the CSF may be activated by infection at that site. That such activation is comparable in the presence or absence of *Cryptococcus* suggests that the local activating infection may be chronic HIV itself or the acute secondary pathogen. Thus, greater B cell differentiation characterizes the circulating B cell populations in HIV infection with or without cryptococcal meningitis infection, with prominent activated phenotypes being overexpressed in the CSF.

### Preferential PD-1 expression on differentiated and activated B cells in blood and CSF.

**FIG 3:**
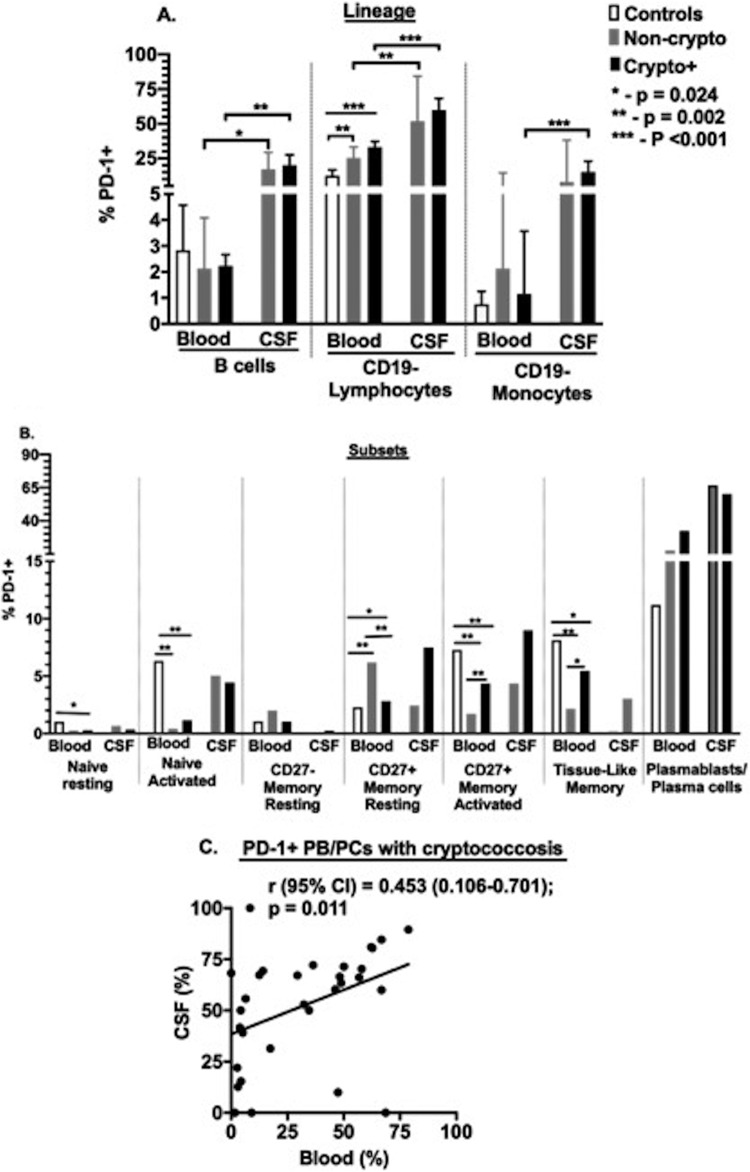
Proportion of PD-1 expression on B cells and non-B cell cellular lineages. Control blood samples (*n* = 10), noncryptococcosis samples (blood [*n* = 7] and CSF [*n* = 7]), and cryptococcosis samples (blood [*n* = 31] and CSF [*n* = 31]). (A) PD-1^+^ expression measured as a frequency of CD19^+^ B cells. (B) Correlation of the frequency of PD-1^+^ expression on plasmablasts/plasma cells in blood and in CSF. (C) PD-1^+^ expression among B cell subsets. Bars show median values. Group values were compared using either Mann-Whitney U test or Kruskal-Wallis test. *, *P* < 0.05.

Programmed death-1 (PD-1) is a surface regulatory “check point” molecule identified prominently on T follicular helper cells and, less frequently, on B cells, NK cells, NKT cells, and other myeloid-derived cells (18). PD-1 was expressed on a minority of circulating B cells but significantly more commonly on B cells in the CSF (Fig. 3A). On CD19^−^ lymphocytes (majority T cells), PD-1 though most prominent in this cell population was more frequent in CSF than in blood (Fig. 3A). On the CD19^−^ monocytes, PD-1 was more frequently expressed in the CSF of cryptococcosis patients than in blood (Fig. 3A). In blood, PD-1 expression was increased on more mature and on activated B cells, a pattern most directly applicable to healthy control subjects and those with cryptococcosis (Fig. 3B). Most striking was the prominent high frequency of PD-1 on circulating CD27^+^ activated memory, tissue-like memory, and plasmablasts/plasma cells in healthy controls and in the cryptococcosis coinfected group (Fig. 3B).

Among other subsets, PD-1 was more commonly expressed on activated naive and CD27^+^ B cells from the blood of healthy control subjects than of HIV-infected adults (Fig. 3B; see also Table S3 in the supplemental material). Among circulating memory B cells from adults with HIV infection, PD-1 was more prevalent on activated memory B cells (CD27^+^ memory activated and tissue-like memory cells) in those with *Cryptococcus* than in those with noncryptococcal meningitis. On the contrary, PD-1 was less frequent on resting CD27^+^ memory B cells in those with *Cryptococcus* than in those with noncryptococcal meningitis.

As in blood, PD-1 in CSF was identified most frequently on PB/PCs. These values were significantly associated in the two compartments among cryptococcosis subjects (Fig. 3C). Thus, the preferential display of PD-1 on activated and differentiated memory B cells in the healthy controls and in those with cryptococcosis invokes the possibility of a regulatory role of this molecule on B cells in cryptococcal infection.

### PD-1^+^ expression on plasmablasts/plasma cells correlates with mortality among cryptococcal meningitis subjects.

**FIG 4:**
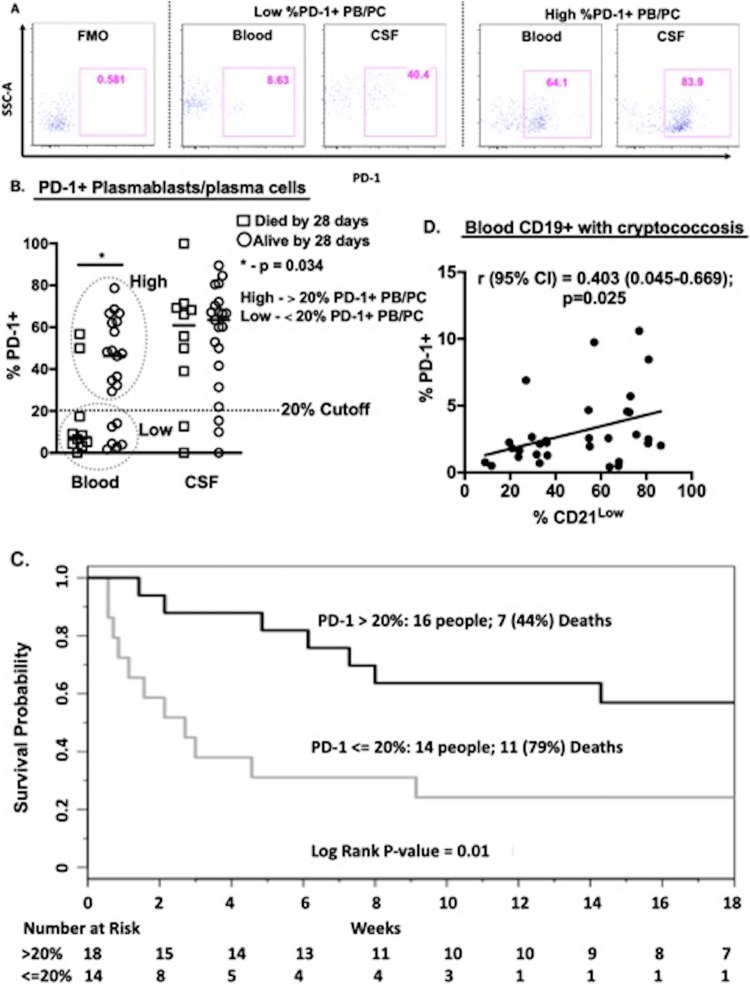
Programmed death-1 expression on blood plasmablast/plasma cells at onset of cryptococcosis predicts 28-day survival or mortality. PD-1, programmed death-1 receptor; PB/PC, plasmablasts/plasma cells; non-CM, HIV and noncryptococcal meningitis coinfection; CM, cryptococcal meningitis. (A) Individual profile of PD-1^+^ PB/PCs among low and high PD-1^+^ PB/PCs. (B) Frequency of PD-1^+^ plasmablasts/plasma cells measured as frequency of PD-1^+^ expressing plasmablasts/plasma cells among cryptococcosis subjects who died by 28 days (*n* = 10) versus survivors at 28 days (*n* = 20). (C) Kaplan-Meier survival outcomes over time among cryptococcal meningitis subjects with low (<20%) (*n* = 14) and high (>20%) (*n* = 16) PD-1 expression on plasmablasts/plasma cells. (D) PD-1^+^ and CD21^low^ expression on B cells. Hazard ratio was determined as log rank of survival days. Three cryptococcosis patients were lost to follow-up after hospital discharge and were excluded from the survival analysis. Mortality was determined at 18 weeks of follow-up.

As noted above, overall mortality at 18 weeks was high in those with cryptococcal meningitis (18/30; 60%). Among the various B cell subsets, only PD-1^+^ expression on circulating plasmablasts/plasma cells was significantly associated with survival (also overall B cell activation by univariate but not multivariate analysis). Of the 10 subjects who died within 28 days after diagnosis with cryptococcosis, PD-1 was identified on a median of 7% of circulating plasmablasts/plasma cells. In sharp contrast, PD-1 was expressed by a median of 46% of these cells among 20 survivors (Fig. 4A and B). Thus, low PD-1 expression was associated with early mortality, and high PD-1 expression was associated with survival.

Using PD-1^+^ plasmablasts/plasma cells as a continuous variable, every 5 units increase in PD-1^+^ expression on these cells was associated with a 17% lower chance of death in the acute setting by 28 days when most mortality occurred (hazard ratio [HR] [95% CI] = 0.83 [0.71 to 0.98]; *P* = 0.02). This association of PD-1 on plasmablast/plasma cells at presentation was no longer significant with overall mortality at 140 days (18 weeks) (HR [95% CI] = 0.93 [0.84 to 1.02]; *P* = 0.13). In further exploration, using a cutoff point of 20% PD-1 plasmablast/plasma cell expression, subjects with PD-1 values of ≤20% had an increased risk of death (log rank *P* value = 0.01) (Fig. 4C). Those with PD-1 values of <20% had a 7-fold increased risk of 28-day mortality compared to those with PD-1 values of >20% (HR [95% CI] = 7.38 [1.69 to 34.36]; *P* = 0.01). While intriguing, the confidence interval is significant but wide and the *P* value is not very small, so this cutoff value is exploratory. By univariant analysis, only circulating B cell activation independently correlated with death (*P* = 0.03) but other reported risks for mortality did not (age, gender, Glasgow coma score, CSF protein, CSF white cell counts, or CSF fungal quantitative culture) (19, 20). PD-1 expression on circulating B cells was associated with B cell activation in blood (Fig. 4D). Whether these observations represent a plausible mechanistic impact of B cells and PD-1 on host survival or a secondary effect is under investigation.

## DISCUSSION

The persistence of very high mortality caused by cryptococcal meningitis during HIV infection in sub-Saharan Africa despite early diagnosis (21) drives efforts to improve antifungal therapy and complementary immune mechanisms of control. We describe in detail for the first time the distinct B cell subset maturation, activation, and regulatory markers in paired CSF and blood from HIV-infected individuals with and without cryptococcal meningitis and associated early mortality. We found a higher population of activated B cells that constituted circulating naive activated, memory activated, and plasmablasts/plasma cells with HIV-associated meningitis that were higher yet in CSF than in blood. In addition, PD-1 expression sequestered more on activated B cell subsets in CSF lineages than in blood. Distinctively, higher PD-1 expression (>20%) on a population of blood plasmablasts/plasma cells correlated with cryptococcosis host survival.

The recognition that cryptococcosis during advanced HIV disease is both systemic and neurologic is supported by the consistently high levels of fungal antigen in both blood and CSF at the time of or preceding clinical diagnosis. The related B cell responses to both systemic and neurologic cryptococcosis are supported by the correlation between B cell subsets, activation, and PD-1 expression in blood and CSF compartments identified herein. The appreciation of how localized infections modulate local responses is demonstrated in the cryptococcosis mouse model of severe and chronic immunodeficiency, lymphocyte adaptive transfer experiments, and B cell knockout models (22, 23). Here, B cells are predominantly important in the control of cryptococcosis brain infection. The CD4 T cells and innate mechanisms are important in control of lung infection while a combination of factors is required to control systemic cryptococcosis. Thus, the overarching importance of the brain and systemic cryptococcosis infection in the human host emphasizes the importance of *Cryptococcus*-specific B cell responses for control. Thus, localized meningitis infections may induce compartmentalized responses with a possibility of a few such responses in circulation. Consequently, the search for biomarkers for compartmentalized infections in circulation in this and other settings may be less informative.

Consistent with earlier reports, circulating B cells from untreated HIV viremic patients show diffuse B cell activation, deficits in memory B cells, and increased representation of tissue-like memory and plasmablast/plasma cells (24–26). The impaired B cell responses due to HIV infection are seen to influence B cell responses in cryptococcosis coinfection (27). This underpins the importance of host immune responses in the primary infection (HIV infection) in determining immunological outcomes of secondary or super infections (cryptococcosis coinfection). In support of this paradigm, our results from HIV-infected patients with low CD4^+^ T cell numbers were consistent with those from HIV coinfected individuals with meningitis due to *Cryptococcus* and other or indeterminate causes. This extends the B cell characterization in these HIV-coinfected subjects. However, we could not determine whether the prominent B cell subset and activation perturbations in blood were due to the chronic effects of advanced HIV disease alone or due to an additional effect of acute secondary infection where cryptococcal antigen and symptoms develop 1 to 4 weeks prior to diagnosis (19, 20, 28, 29). This distinction is important in determining whether patients with such advanced disease can actually generate a specific response to this systemic and local neurologic cryptococcal infection. The fact that T cells to the fungus are detected in blood (30) and antibodies in blood (31, 32) and CSF (E. Finn, S. Okurut, and E. N. Janoff, unpublished data) suggests that they can.

In this context, these data build on a limited but well-derived set of observations about the presence of B cells in the CSF during HIV infection. The predominance of T cells is observed in the CSF of HIV-infected subjects with and without *Cryptococcus* (33–35). Among HIV-1-infected adults without neurologic disease, uncharacterized B cells represented a small proportion (≈1%) of lymphocytes in CSF, albeit more frequent than in heathy control subjects (34). We found that B cells in the CSF represented a median of 2.3% or 2.6% of lymphocytes with cryptococcal meningitis or meningitis of other origin, respectively. The CSF B cells in our population were distinguished by prominent activation (median, 77% and 68% CD21^−^ B cells), majority memory and tissue-like memory phenotype, and high numbers of plasmablast/plasma cells (median, 13% and 8% in the two meningitis groups).

Akin to persons with multiple sclerosis (36, 37), short-lived CSF plasmablasts were reported to be increased in adults with HIV infection, particularly early in their course (38). The fact that B cells and plasmablast frequencies in the CSF correlated with HIV RNA in CSF and decreased with antiretroviral therapy (ART) in that study implicated the virus as a stimulus for the presence of these cells. The B cells and short-lived plasmablasts were also increased with neurosyphilis and declined with therapy (39), highlighting that secondary infections can elicit these cells. We could not distinguish between the more prominent plasmablasts and infrequent plasma cells described with our markers, but the increased frequency of B cells of memory and plasmablast/plasma cells with meningitis is consistent with the CNS responses to neurosyphilis and viral meningitis and in multiple sclerosis (36–41). Whether ectopic germinal centers are present in the brain with cryptococcal meningitis, which has a prominent component in the brain parenchyma, as were identified with neurosyphilis (39) as a source of local B cell generation for antibody production, is under investigation.

A distinctive feature of B cells in both blood and CSF in this study was the prominent expression of the checkpoint regulatory marker PD-1. PD-1 (CD279) is well recognized as a coinhibitory molecule, particularly during chronic viral infection, e.g., with HIV, where PD-1 expression is associated with CD8^+^ T cell exhaustion, low T cell proliferative capacity, defects in effector T cell function, and HIV disease progression (42). In addition to CD4^+^ and CD8^+^ T cells, PD-1 is expressed on activated NK T cells, monocytes, and B cells. As an immunomodulatory surface receptor, PD-1 on B cells can downregulate responses elicited through antigen-specific B cell receptors (BCRs) by dephosphorylating key cytoplasmic B cell signal transducers (43). The PD-1 on CD4^+^ T follicular helper cells supports reversible inhibition of B cell responses via PD-1 interaction with its ligand PD-L1 on B cells during HIV infection (25). However, PD-1 on B cells has diverse and potent effects on B cell function with systemic impact. Among our subjects with cryptococcal and other forms of meningitis, PD-1 expression was increased on activated B cells, particularly CD27^+^ and tissue-like memory cells, as reported by others (42, 44). Indeed, microbial antigens, modeled by Toll-like receptor-9 agonists, augment PD-1 expression on human B cells together with IL-10 production (45).

Similar to PD-1 effects on T cells, PD-1 on B cells can inhibit B cell activation, suppress B cell proliferation, and impair B cell inflammatory cytokine responses (12, 13, 44, 46). Although less prominent on human B cells, PD-1 has been described at high levels among simian immunodeficiency virus (SIV)-infected macaques in association with loss of memory B cells and fatal intestinal infection (47). The prominence of PD-1 on CSF B cells in our patients with cryptococcal meningitis and the association of PD-1 expression with mortality may be consistent with the results of PD-1 expression in the SIV primate model of macaques that also died with secondary infections. Blockade of PD-1 in the SIV macaque model augmented SIV-specific antibodies and improved survival. Also, the PD-1/PD-L1 axis antagonists suppressed immune activation and inflammation that resulted in limited microbial translocation across the gut mucosa associated with the improved integrity of the tight junctions lining gut mucosa (48, 49). Interestingly, the PD-1 kinetics model on T cells shows that PD-1 immune modulatory mechanisms work independent of immune status. The PD-1 immune modulated responses on T cells are demonstrated to be consistent in a macaque model of intact immune system with those in the same host with induced immune suppression with SIV infection (49). Thus, PD-1 modulation in human cryptococcosis may influence cryptococcal transmission across the blood-brain barrier to influence onset and extent of pathology.

The association of PD-1 expression on circulating B cells with cryptococcosis host survival suggests that this immunomodulatory protein may synergize B cell functions to control *Cryptococcus* infection. However, whether the underlying mechanisms depend on the presence or absence of the PD-1 ligand PD-L1 or other factors involved or resulting from PD-1/PD-L1 interaction is an area of investigation. In the mouse model of cryptococcosis, *Cryptococcus* upregulates PD-1 expression to maintain host tolerance to *Cryptococcus* infection, as may be the case in autoimmune diseases (50, 51). Moreover, the altered PD-1 role with PD-1 antibody blocker in mouse cryptococcosis infection improved host survival. The improved survival was associated with the downregulation of immune regulatory responses (IL-10 and IL-5), enhanced expression of proinflammatory cytokine responses (Th1/Th17 balance), and increased fungal clearance (52). In summary, the role of PD-1 expression and altered cellular functions are demonstrated to be similar in a spectrum of cells in diseased hosts. Thus, high PD-1 expression could confer reduced host damage response benefits at the expense of unchecked fungal replication that could be effectively managed with optimal antifungals. Hence, PD-1 expression could be an important marker for determining clinical phenotype, disease progression, or host recovery with antifungal treatment. We propose that understanding the mechanism of PD-1 induction and characterizing the consequent immune and genetic determinants of disease progression or recovery with optimal antifungals in this setting will advance our understanding of the multiple facets of host immune dysfunction for translation.

## MATERIALS AND METHODS

### Study participants.

HIV-infected adults in Kampala, Uganda, with cryptococcal meningitis (cryptococcosis) (*n* = 31) and with noncryptococcal meningitis (noncryptococcosis) (*n* = 12) were selected from the following three prospective HIV meningitis clinical cohort studies: (i) Cryptococcal Optimal Antiretroviral Timing (COAT) trial (20) (ClinicalTrials.gov identifier NCT01075152), (ii) Neurological Outcomes on Antiretroviral Therapy (NOAT) study (53), and (iii) Adjunctive Sertraline for the Treatment of HIV Associated Cryptococcal Meningitis (ASTRO) trial (ClinicalTrials.gov identifier NCT01802385) (54). Inclusion criteria were as follows: clinical evidence of meningitis, age of ≥18 years, documented HIV infection, not receiving ART at enrollment, and availability of cryopreserved cells from blood and CSF (Table 1). HIV infection was confirmed by bedside testing of previously undiagnosed subjects using the Ugandan Ministry of Health/WHO HIV testing algorithm. Cryptococcosis was confirmed by the presence of cryptococcal antigen by lateral flow assay (Immy Inc., Norman, OK, USA) in CSF and CSF cryptococcal quantitative cultures (55). The noncryptococcal meningitis subjects were confirmed with tuberculosis and rifampin GeneXpert, 16S rRNA, and by quantitative fungal and bacterial culture as previously described in this HIV meningitis cohort (53). Of the noncryptococcal-meningitis-coinfected subjects, 4 had Mycobacterium tuberculosis meningitis; 1 had neurosyphilis, toxoplasmosis, and Mycobacterium tuberculosis coinfections; and 7 were without a known HIV coinfecting etiology. Whole blood, but not CSF, was obtained from healthy control subjects with neither HIV nor cryptococcal infection (*n* = 10) from an HIV observational rural Ugandan cohort (56). Subjects prospectively provided informed written consent for the parent studies. Makerere University Research Ethics Committee granted ethical approval for use of stored specimens from previously consenting adults. None of the subjects was on steroids, on ART, or on antifungal therapy prior to sample collection.

### Sample preparation.

Peripheral blood mononuclear cells (PBMCs) (blood) and CSF samples were collected within 72 h of meningitis diagnosis. Blood and CSF cells were isolated and cryopreserved as previously described (33) in Roswell Park Memorial Institute enriched medium (69%) supplemented with 20% fetal bovine serum, 10% dimethyl sulfoxide, and 1% penicillin-streptomycin in vapor phase in liquid nitrogen until testing. Blood and CSF cells were thawed, and cell viabilities and cell recoveries were determined using an automated Guava PCA instrument before antibody staining for flow cytometry.

After thawing, median cell viability was 91% (range, 75 to 98%) from the CSF and 98% (range, 95 to 100%) from frozen PBMCs. Median cell recovery was 1.2 × 10^6^ cells (range, 0.1 × 10^6^ to 5 × 10^6^ cells) per subject from CSF and 8 × 10^6^ cells (range, 3 × 10^6^ to 25 × 10^6^ cells) per subject from blood.

### Immunophenotyping.

**FIG 5:**
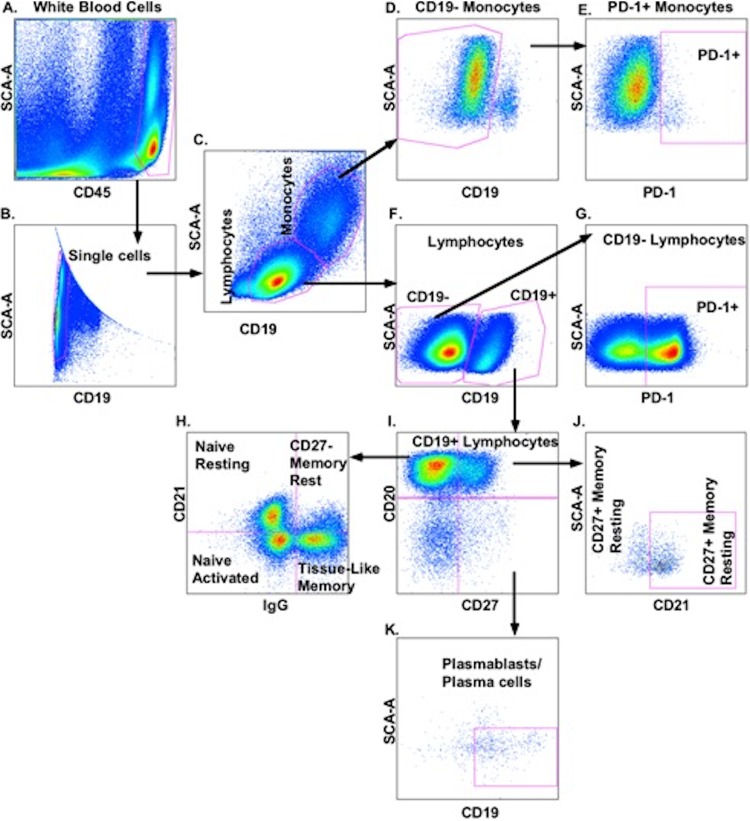
B cell gating strategy used to characterize activation and cellular differentiation in blood and CSF illustrated entirely on blood. Leukocytes are distinguished by expression of CD45^+^ expression (A) to exclude cryptococcal yeasts and selected for single lymphocytes (B and C). PD-1 is gated on CD19^−^ monocytes (D and E) and CD19^−^ lymphocytes (F and G). B cell subsets (F to K) are defined per Table S1 in the supplemental material as described earlier (14). Gates indicated for PD-1, CD21, and CD38 were determined using fluorescence minus one cut-off (not shown) to define subset expression, activation, and PD-1 expression.

Thawed blood and CSF cells were stained with murine monoclonal antibodies reactive with human CD45 (fluorescein isothiocyanate [FITC]; clone HI30), CD20 (allophycocyanin [APC]-Cy7; clone 2H7), and CD38 (phycoerythrin [PE]-Cy7; clone HIT2) (BioLegend, San Diego, CA); CD19 (V500; clone HIB19) (BD Horizon, San Jose, CA); CD27 (peridinin chlorophyll protein [PerCP]-Cy5.5; clone M-T271), PD-1 (PE; clone EH12.1), and IgG (APC; clone G18-145) (BD Pharmingen, San Jose, CA); and CD21 (Pacific Blue; clone LT21; EXBIO Praha a.s., Czech Republic). CD45 expression was used to discriminate white blood cells in CSF and in blood from *Cryptococcus* yeast cells. Data were acquired using BD FACSCanto II and an 8-color flow cytometer with BD Diva software (BD Bioscience San Jose, CA, USA) and analyzed using FlowJo version 9.7.7 (Tree Star, Ashland, OR, USA). Gating was established for CD21, IgG, PD-1, CD38, CD20, and CD27 expression using fluorescence minus one control. Spectral overlap was compensated using BD FACS compensation positive mouse Igκ beads and BD FACS compensation negative mouse Igκ beads (BD Biosciences, San Jose, CA, USA). A representative gating scheme is shown in Fig. 5.

### B cell differentiation and activation.

Based on results in Fig. 5 and designations in Table S1 in the supplemental material, CD45^+^ CD19^+^ lymphocytes were characterized as resting or activated (CD21^+^ versus CD21^−^, respectively) naive (CD20^+^/CD27^−^/IgG^−^), memory (CD27^+^ or CD27^−^/CD20^+^ IgG^+^ CD38^−^), or tissue-like memory (CD27^−^/CD21^low^/IgG^+^) B cells and plasmablasts/plasma cells (PB/PCs) (CD20^−^ CD27^++^/CD38^++^/CD21^low^). Combined subsets accounted for 97.8% to 98.3% of gated CD19^+^ B cells in blood and 67.9% to 82.0% in the CSF in each group.

### Statistical analysis.

Data were analyzed using GraphPad Prism for Macintosh version 8.0 (San Diego, CA, USA). Nonparametric Wilcoxon signed-rank test analyzed paired continuous variables, Mann-Whitney U test and Kruskal-Wallis tests analyzed unpaired continuous variables, and Kruskal-Wallis test and analysis of variance (ANOVA) analyzed three-group data. Survival was summarized with Kaplan-Meier plots and compared between groups with a log rank test. Proportional hazards regression models were used to quantify the risk of death between groups. Survival data were censored at time of death, loss to follow-up, or at 18-weeks (the minimum follow-up time for the three studies). A *P* value of ≤0.050 was considered statistically significant.

## Supplementary Material

Supplemental file 1
