# Supplementary material for: B Cell Compartmentalization in Blood and Cerebrospinal Fluid of HIV-Infected Ugandans with Cryptococcal Meningitis
Source: Infect Immun. 2020 Feb 20;88(3):e00779-19. doi: 10.1128/IAI.00779-19 (PMC7035924; doi:10.1128/IAI.00779-19)
Supplement: Supplemental file 1 [file zii999093021s1.pdf]

Supplementary Table S1. Markers of B cell subsets and activation

B cell phenotypes	CD45	CD19	CD20	CD27	IgG	CD21	CD38	PD-1
**Naïve resting**	+	+	+	-	-	+	-	+/-
**Naïve activated**	+	+	+	-	-	-	-	+/-
**CD27- Memory Resting**	+	+	+	-	+	+	-	+/-
**Tissue-Like Memory**	+	+	+	-	+	-	-	+/-
**CD27+ Memory resting**	+	+	+	+	+/-	+	-	+/-
**CD27+ Memory activated**	+	+	+	+	+/-	-	-	+/-
**Plasmablast/ Plasma cells**	+	+	-	+	+/-	-	+	+/-

Positive (+) shows presence of marker expression, negative (–) shows absence of marker expression and (+/-) show either presence or absence of marker expression.

Supplementary Table S2. B cell subsets and activation in cerebrospinal fluid (CSF) and blood among subjects with HIV-1 infection and cryptococcal and non-cryptococcal meningitis

**Study group,**	**Blood**	**CSF**	**P value**	**Blood**	**CSF**	**P value**
B cell subsets	CM	CM		Non-CM	Non-CM	
n	31	31		7	7	
Naïve resting	35.8 (18.9-60.5)	2.6 (0.7-8.6)	**<0.001**	16 (10.9-39.9)	2.6 (0.8-13.2)	
Naïve activated	28.4 (15.6-38.3)	15.2 (8.5-22.8)	**0.007**	53.1 (43.1-83.4)	68.4 (57.5-75.3)	
CD27- Memory resting	1.4 (0.4-3.2)	1.9 (0.3-4.5)		2.3 (0.9-29.5)	5.1 (2.9-11.9)	
Tissue-like memory	9.1 (3.4-20.2)	7.9 (4.4-12.2)	**0.001**	20.3 (6.5-22.4)	9.1 (4.3-18.5)	
CD27+ Memory resting	3.5 (1.2-5.4)	8.5 (3.4-13.9)	**0.001**	5.2 (0.8-11.4)	11.3 (8.7-22.4)	
CD27+ Memory resting	4.9 (2.0-9.3)	27.2 (14.3-39.9)	**<0.001**	5.9 (2.7-17.7)	24.7 (21.1-33.3)	

Plasma blasts/ Plasma cells	0.7 (0.2-2.4)	13 (3.3-20.9)	**<0.001**	0.9 (0.6-1.3)	8.5 (4.4-14.2)	**0.008**

All values as shown are median (IQR) percent of CD19+ B cells. P values were calculated with either Wilcoxon paired t-test; (Within cryptococcosis infected individuals: CSF vs. Blood comparisons) or Mann-Whitney unpaired U-test; (cryptococcosis vs. Non-cryptococcosis).

Supplementary Table S3. Programmed Death-1 (PD-1+) Expression on B Cell Subsets in CSF and in Blood with HIV infection with and without Cryptococcal Meningitis

Study group, PD-1+ B cells	Blood, CM	CSF, CM	P value	Blood, Non-CM	CSF, Non-CM	P value
**n**	**31**	**31**		**7**	**7**	
**Naïve resting**	0.3 (0.2-1.0)	0.3 (0-2.6)		0.2 (0.1-2.0)	0.6 (0-2.2)	
**Naïve activated**	1.1 (0.7-1.9)	4.4 (1.4-7.7)	**<0.001**	0.4 (0.2-1.3)	5.0 (1.3-16.5)	
**CD27- Memory resting**	1.0 (0.6-3.6)	0.2 (0-4.1)		1.9 (0-8.6)	0 (0-0.5)	**0.009**
**Tissue-like memory**	5.4 (3.0-8.0)	3.0 (0.5-4.8)	**0.003**	2.2 (1.1-3.3)	0.1 (0-1.8)	
**CD27+ Memory resting**	2.8 (1.0-4.9)	7.5 (2.5-17.8)	**0.001**	6.2 (3.1-14.6)	2.4 (0-20.0)	
**CD27+ Memory activated**	4.4 (2.4-6.7)	9.0 (4.4-17.5)	**0.001**	1.7 (0.8-3.5)	4.4 (0-21.3)	
**Plasmablasts/ Plasma cells**	32.3 (5.2-56.8)	60.2 (31.4-70.3)	**<0.001**	17.6 (5.9-26.1)	66.7 (44.5-100)	**0.026**

All values as shown are medians (IQR) and percent of parent (B cells subsets). P values were calculated with either Wilcoxon paired t-test; (within Cryptococcosis subjects; CSF Vs. Blood comparisons or Mann-Whitey unpaired U-test among non-cryptococcosis subjects Vs. Non-cryptococcosis subjects.
